# Supplementary material for: Surround suppression in mouse auditory cortex underlies auditory edge detection
Source: PLoS Comput Biol. 2023 Jan 19;19(1):e1010861. doi: 10.1371/journal.pcbi.1010861 (PMC9888713; doi:10.1371/journal.pcbi.1010861)
Supplement: S1 Fig — a. A scheme showing the number of SUs outputted by the spike sorting procedure (n = 577), the number of neurons responsive to one of various attributes of sounds (n = 451), highly responsive units to BBSs (n = 149), and the number of neurons with high fit-correlations (above median) to the MRW model (n = 74). see Methods for further information. (PDF) [file pcbi.1010861.s001.pdf]

Supplementary Figure 1

a.

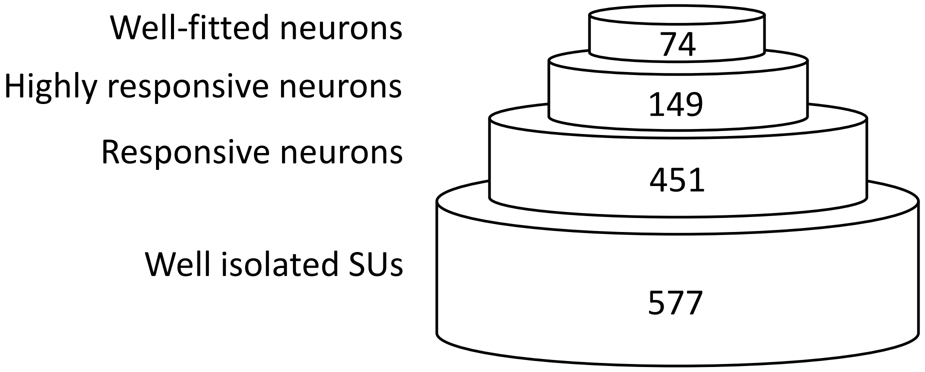

<u>Supplemental Figure 1 – neuronal counts</u>

a. A scheme showing the number of SUs outputted by the spike sorting procedure (n=577), the number of neurons responsive to one of various attributes of sounds (n=451), highly responsive units to BBSs (n=149), and the number of neurons with high fit-correlations (above median) to the MRW model (n=74). see Methods for further information.
