# Supplementary material for: Surround suppression in mouse auditory cortex underlies auditory edge detection
Source: PLoS Comput Biol. 2023 Jan 19;19(1):e1010861. doi: 10.1371/journal.pcbi.1010861 (PMC9888713; doi:10.1371/journal.pcbi.1010861)
Supplement: S2 Fig — a. Schematic of the calculation of a response of a model neurons with a given tuning curve (middle) to an example BBS modeled with cochlear widening (right). The inner product of the tuning curve and the stimulus is then positively rectified. b. Example notch stimulus, modeled with cochlear widening. (PDF) [file pcbi.1010861.s002.pdf]

Supplementary Figure 2

a.

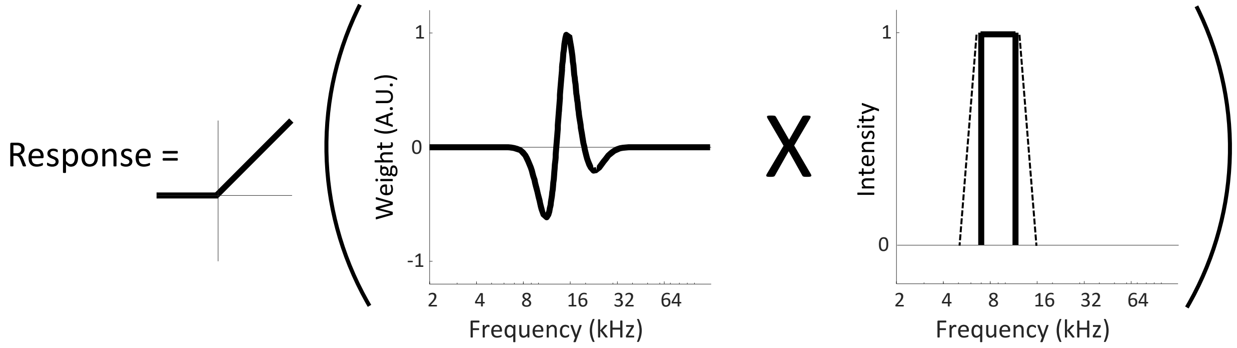

b.

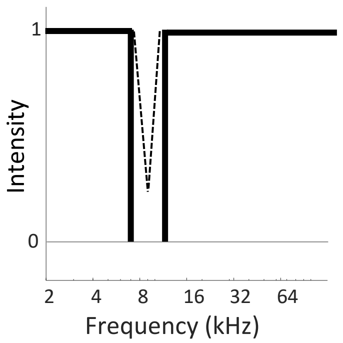

<u>Supplemental Figure 2 – Response function</u>

a. Schematic of the calculation of a response of a model neurons with a given tuning curve (middle) to an example BBS modeled with cochlear widening (right). The inner product of the tuning curve and the stimulus is then positively rectified.

b. Example notch stimulus, modeled with cochlear widening.
