# Supplementary material for: Surround suppression in mouse auditory cortex underlies auditory edge detection
Source: PLoS Comput Biol. 2023 Jan 19;19(1):e1010861. doi: 10.1371/journal.pcbi.1010861 (PMC9888713; doi:10.1371/journal.pcbi.1010861)
Supplement: S3 Fig — a. Top: FBRAs of 4 neurons from 3 mice. Middle: model FBRAs fitted to the same neurons. Bottom: fitted MRWs of the same neurons. (PDF) [file pcbi.1010861.s003.pdf]

Supplementary Figure 3

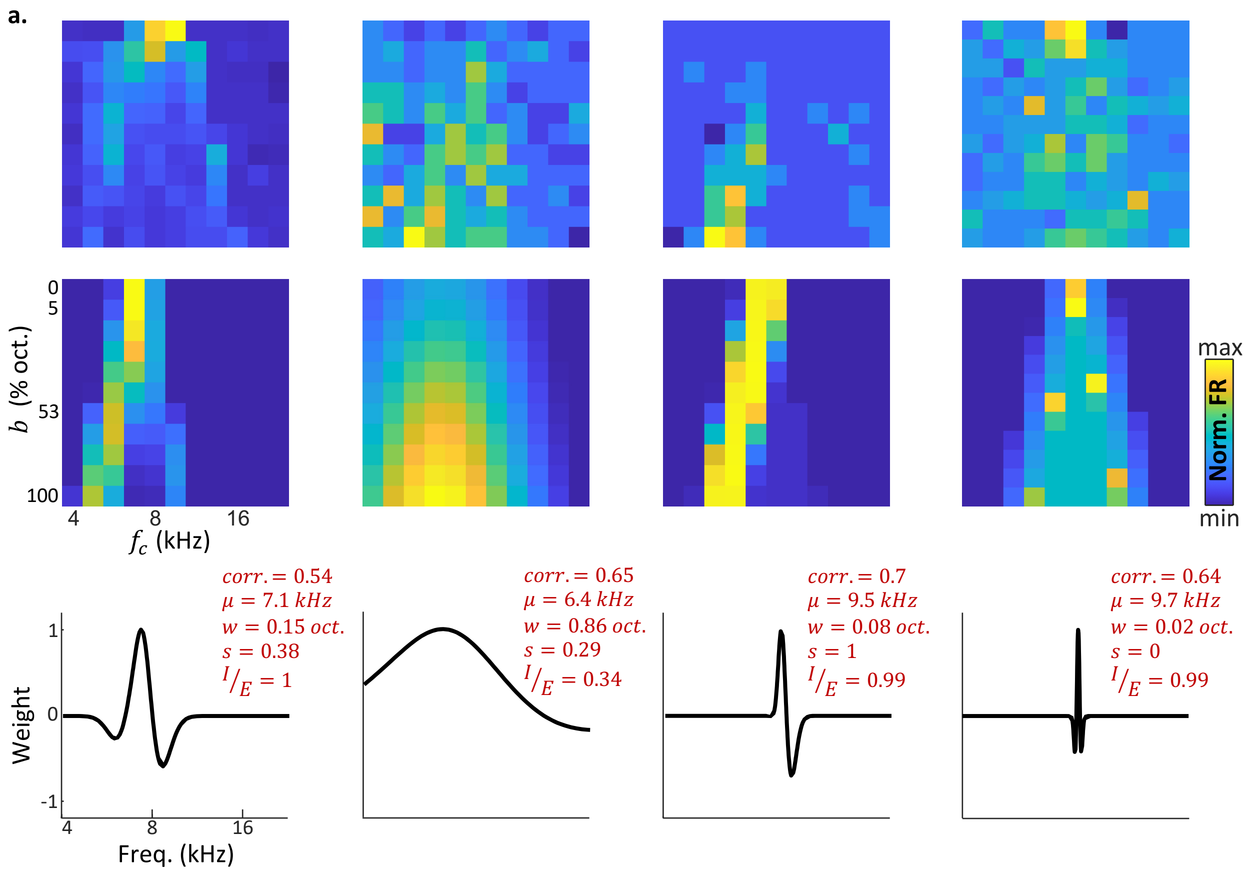

Supplemental Figure 3 – Additional examples of model fitting

a. Top: FBRAs of 4 neurons from 3 mice. Middle: model FBRAs fitted to the same neurons. Bottom: fitted MRWs of the same neurons.
