# Supplementary material for: Surround suppression in mouse auditory cortex underlies auditory edge detection
Source: PLoS Comput Biol. 2023 Jan 19;19(1):e1010861. doi: 10.1371/journal.pcbi.1010861 (PMC9888713; doi:10.1371/journal.pcbi.1010861)
Supplement: Attachment — Submitted filename: Gilday_Rebuttal.docx [file pcbi.1010861.s006.docx]

**Answers to Reviewers (manuscript # PCOMBIOL-D-22-01350)**

We thank the editor and reviewers for their constructive comments. We apologize for the delay in responding, which stemmed from the fact that the first author of the paper is no longer in the lab, and from teaching chores. We replied to all comments below (answers in blue). The new text in the revised manuscript appear as **bold** text.

----

Editors comments

*Dear Adi and co-authors,
I hope that you will find the feedback of two expert reviewers useful for your revised manuscript. I have also found your paper quite interesting and well done. The two populations of neurons that you found using this approach is intriguing as well. At this stage of the review, I have just two major comment and a few minor ones.*
Answer: Thanks for these comments.

*My first major comment is that in the limitations of your study you never mention that you have not attempted to generalize it to stimuli beyond short static (and “non-natural” ) stimuli. If your description of the “tuning” of the neuron is correct it should be able to predict time-varying responses to natural sounds as the mouse experiences it in its environment. I agree with you that a family of broad band stimuli is better than short pure tones or simple combination of tones but it is still quite a restricted stimulus. You should at least discuss these limitations.*
Answer: We now added this limitation to the discussion (see p. 13).

*My second major comment is that you are effective obtaining static spectral receptive fields using a parametrized stimulus. You briefly mention the limitation of ignoring the temporal dimension (citations 57, 69) but you might also state that inhibitory side bands have been reported before by estimating spectral temporal receptive fields (STRFS) both in mammals and in birds. In these studies complex broad band sounds with spatial temporal structure are used in combination with reverse correlation techniques. One could argue that that “older” approach is even better? Some missing relevant citations: Qiu A, Schreiner and Escabi J. Neurophys 2003; Miller et al. J. Neurophys 2002; Depireux et al. 2001; Woolley et al. J. Neuroscience 2009.*
Answer: We now mention that inhibitory sidebands were shown using STRFs and cite the relevant papers (see pp. 12-13).

*Minor:
1. Top of p.6: When describing the stimuli and the effect of increasing the bandwidth, I would say that the signal rms increased. If you talk about sound intensity, you would have to define what you mean by intensity and clearly distinguish it from sound loudness.*
Answer: Done (p. 7).

*2. Bottom of p.7. You say: “These data show that our model has a strong descriptive power over the electrophysiological data.” You are actually make correlations between one model - (model FBRA) - and one summary statistic of your ephys data - emperical FBRA. You are not predicting time-varying responses or the occurrence of single spikes. Even for 100 ms long static stimuli, there is clearly some dynamics in the response.*
Answer: We changed the text to: “*These data show that our model has a strong descriptive power for estimating the mean responses of ACx neurons to BBS*” (p.8).

*3. The notch stimuli are a good control. To be candid and really generalize, one might have also used short natural stimuli (100 ms) and in a different classes of sounds all together such as mouse song.*
Answer: We agree. But this is beyond the scope of this paper, as we now mention in the Discussion (p.13).

*4. It might be fine to use the default values in kilosort for selecting SU but I would state more explicitly what algorithm was used and with what parameters. The central vs shoulder part of the auto-correlation function that is estimated in those algorithms to assess refractory violations might not be appropriate for your data.*
Answer: We used kilosort 2.0 with its default parameters, which is a benchmark software  (Pachitariu et al., 2016, Feigin et al., 2021). The central part of the autocorrelation was estimated with a refractory period of 2ms (Berry & Meister, 1997; Cheng et al., 2006). The shoulder part of the autocorrelation was estimated through the baseline firing rate of the respective cluster. These parameters were shown to be appropriate for neocortical SUs, as shown for example by Steinmetz et al., 2019.  In addition, the algorithm classification was further evaluated manually when each suggested SU inspected in criteria of waveform size, consistency, and the presence of short-latency ISIs. The process is now stated with further details in the Methods section (p. 17).

*Reviewer's Responses to Questions*

***Comments to the Authors:
Please note here if the review is uploaded as an attachment.***

*Reviewer #1: 
The manuscript entitled ‘Surround Suppression in Mouse Auditory Cortex Underlies Auditory Edge Detection’ is truly very interesting. The authors record from the auditory cortex of awake mice using Neuropixels probes and a novel and very elegant sound stimulation protocol. The combination with modelling, allows them to explore the sensitivity of auditory neurons to the frequency bandwidth of the stimulus. The authors find that neurons are sensitive to the bandwidth of the sound stimulation, that this sensitivity is evidenced through surround suppression, and that the asymmetry of this side inhibition results in the coding of upper frequency edges. Surround suppression and edge detection are phenomena well explored in other modalities, notably in vision, and implied in auditory data. The current protocol presents evidence for edge detection in the auditory cortex and opens a door to study this dimension of auditory processing. The experiment is well designed, and the interpretation of the results is justified, although I miss details in the analysis of edge detection. The manuscript is well written, very clear, and the figures generally appropriate, although see comments below. I found the videos a wonderful contribution to the clarity.*
Answer: Thanks for these comments.

*Main comments:
1) Edge detection is introduced only in Figure 5 and then hardly explored. The shift leftwards in tuning with increased bandwidth is evident already in Figure 1 and leaves the reader wondering why the relationship between bandwidth (and edges) is not explored in more detailed there. The edge detection could be brought forward, already to figure 2 or 3. I think the authors should decide. If they want to leave edge detection to Figure 5, it would help the reader to have, in Figure 1, some indication of how the bandwidth relates to the frequency shift, for example by illustrating for one fc the frequency ranges that are covered with each bandwidth. Then, in figure 5, a transition between the FBRAs and the tuning according to ft is necessary. For example one could rearrange the FBRAs such that rows are ft.*
Answer: We have structured the narrative of the paper such that the interpretation of the data builds **towards** edge detection (i.e. *1. FBRAs → 2. Explaining FBRA with MRW → 3. MRW analysis now gives intuition about edge detection*).
Edge detection could potentially be brought forward to figure 4 but we still think that the model validation should be presented before making use of the conclusions of the model. Also note that since the steps in fc and bandwidth are not consistent and there are no two stimuli with the exact same ft, we cannot create an FBRA aligned with ft. Such a depiction would look something like the graph below. We think that this may be more confusing to the reader, especially as figure 1 is quite crowded as it is. However, if the editor or reviewer insists that such a graphic would clarify the stimulus set, we can definitely add it.

*2) I miss a more thorough analysis of the relationship between the 2 variables identified as more informative, ‘s’ and ‘I/E’, and the other variables. This could include a quantification of the distribution of responses-patterns. I miss more examples of neurons with low model correlation, or at least at the edge of the threshold.*
Answer: First, below we plot the mean and width related to ‘S’ and ‘I/E’. It is difficult to interpret these scatter plots in the context of our findings as two significant correlations (the two central scatters) are mainly a result of neurons with no inhibitory sidebands. If the editor or reviewer think we should include this (perhaps as supplemental figure), we will gladly add these.

Second, as per the reviewer’s request, we now added new responses of neurons that show low correlation with the model (see new Supplemental figure 3 and accompanying text, p. 8).


*3) I wonder how the distribution of edge frequencies in the population contributes to the tonotopic map. Would a tonotopic map centered built with responses to pure tones, look very different form one built with top edge frequencies. If so, how? If the recordings do not span a wide enough range of the tonotopic map, the authors could discuss the issue.*
Answer: We do not know how to answer this because we did not carefully map tonotopy, and the range of BF values that we measured is rather narrow and skewed. See Discussion (p. 13).

*Minor comments:
-Line numbers are missing*
Answer: done.
*Abstract:
-What is the question?*
Answer: This is now added to the abstract.

*Methods:
-Were spikes quantified only as ‘the number of spikes during stimulus presentation (100
ms)’?*
Answer: For deciding whether a neuron was significantly active or not, we counted spikes before and after stimulus offset. For FBRAs we quantified spikes in firing rates.

*-In the SU validation, what is the minimum mean spike amplitude, number of spikes per protocol, ISI?*
Answer: In SU validation, we only selected units that were classified as 'good' by the kilosort2 algorithm, by using its default parameters. The threshold which we applied to the template projections (not to the voltage), was set to 10 and the number of spikes to 0.02 per second (see <https://github.com/MouseLand/Kilosort>). The minimal ISI was set to 2 ms.

*Results:
-In most examples, throughout the manuscript, sounds with zero bandwidth elicit very weak responses. I am not sure if these sounds are what is described as pure tones in the methods and, therefore, are sounds of lower intensity? If so, are they necessary in the plots? An exception is the one shown in Figure 2a top right, which also happens to not show responses at increasing bandwidth. Please comment.*
Answer: Yes, zero bandwidth sounds are pure tones. In order to be faithful to the increase in sound intensity along with bandwidth, PT indeed had low intensity (~55 dB SPL). We do feel, however, that in some neurons (like the one mentioned by the reviewer), keeping the response to PT has merits. First, this shows another limitation of our relatively simple model at very low bandwidths (now discussed in the limitation section of the Discussion). Second, as mentioned by the reviewer, the top right neuron in Fig 2a is strongly responsive to PT, even more than to broadband stimuli, which is an anecdotal example of a strong surround suppression (this is now added to the text as well).

*-Figure 1, the asymmetrical FBRAs have a stepwise pattern. Is this because of a difference between the resolution of the fc (plotted) and the frequency range increase with bandwidth?*
Answer: Yes. A single step in the central frequency is 0.25 octave and a single step in the bandwidth is ~0.1%. See p.18.

*-Figure 3, examples of low correlation neurons?*
Answer: Done. See new Supplemental figure 3.

*-Figure 3c is not very informative since neuron clusters fall on a very small region of 1 or 2 quadrants. A histogram of the projection of the each of the 2 axes in Figure 3b would be better in my view. In fact, reading further, I would move Sup. Fig 3 to the main figures. See also main comment 2 above. I like that the dots with -1 I/E values fall on the left of the ordinate, as if they were embarrassed.*
Answer: As suggested, we now moved former S3 into the main figures (See new Fig. 3C). However, we still left the former 3c (now appears as 3d) as it conveys a deeper message. We think that presenting both increases clarity.

*-Was there any correlation between ‘s’ or ‘w’ values and best frequency. Do either of them increase or decrease with BF? The distribution is very skewed so probably not.*
Answer: See answer to main comment #3.

*-What is the value of the population median in ‘we focused on SUs that had the highest correlations with the model, defined as being above the population median’? I could not find it. Since the distribution of correlations is flat, what does this threshold mean?*
Answer: The value of the median is 0.644. This is now stated explicitly in the legend of figure 3. When trying to say something meaningful about the fit parameters, we wanted to avoid treating neurons that had a poor fit, since the choice of parameters for them could have been an arbitrary settlement by the fitting function. Thus, to be fair, we have chosen the median as the simplest heuristic for a threshold for neurons with a meaningful fit.


*-Figure 4e, the fit might be strong but most of the nFBRAs are very close to zero and only as the FBRA becomes 0.6 or above does the nFBRA start to increase. Which brings me back to the question of what is a meaningful threshold for the FBRA correlation. I did not find this analysis very useful. Also, when one then reads about the edge detection in the next section, one is left wondering how the notch relates to this.*
Answer: Figure 4e relates and follows 4d. The purpose of Fig 4d is to show that the nFBRA correlations are skewed to the right, and better than chance. Fig 4e then shows that, as expected, neurons for which nFBRA correlation was high, are those for which FBRA correlation was high to begin with.
nFBRA correlations are high for neurons with FBRA correlation above ~0.6 which is fairly close to the population median, further justifying our choice as a threshold for a neuron to be considered “well-fitted”.
Indeed, the edge-detection aspect of the paper is not directly linked to the notches. The notches, however, provide a nice validation to the model, which is then used in the aspect of the paper dealing with top frequency.

*-Figure 5c. It would help the explanation, and to keep in line with the message of 5a and 5b, if, in addition to the FBRA and tuning curves, there would be also an FBRA aligned by the top, rather than current, frequency. Maybe a video, showing how the tuning changes as the FBRA shifts alignment from fc to ft?*
Answer: See answer to main comment #1.

*-Figure 5d is interesting but I would like to see a correlation between Rt and other variables found of meaning before, such as ‘s’ and ‘I/E’, or the model correlation. If pure tone responses were comparable, it would be interesting to see the relationship between Rt and R pure tone.*
Answer: We expect Rt to be high for neurons that have both high s, and high I/E values (which are the ‘Q1’ neurons), which is why we separated them from the rest of the population. We do not see how to interpret a correlation between Rt and s or I/E separately. For example, a highly skewed neuron with no inhibition will not be selective of top frequency, while a perhaps less skewed neuron with strong inhibition will.

*-While the responses of a neuron to a given set of bandwidths might be the same when these are aligned by upper edge frequency, the maximum response will be to a more centered frequency (t would be nice to see examples of this on the manuscript). Is this because these centered frequencies are played at higher intensities? Or does this reflect a combined sensitivity to edge and center.*
Answer: We did not fully understand this critique.

*Discussion:
-First paragraph: rephrase? It seems to go back and forth. The group of sentences starting ‘Importantly,’ seems to repeat the previous ones. Also, the sentence starting ‘Notably’ is, I think misleading. I did actually miss the analysis of relationship of effects with center frequency…*
Answer: We rephrased the group of sentences starting with “importantly”. Notice that in the beginning of the discussion we state the non-monotonic response profile to bandwidth given a single central frequency has been shown before, and only then we mention that by playing many central frequencies we can see that the bandwidth with peak response is different for different frequencies.
In the sentence starting with “notably” we mention that the optimal bandwidth for each center frequency is not random but follows various patterns. Our analysis of that is actually applying the MRW to the population and quantifying its parameters to explain these regularities (see p. 11).

*-Second paragraph: I appreciate the humbleness, but did you really mean ‘has few merits’? or rather ‘has a few merits’? Same, but less humble, with the limitations.*
Answer: Done.

*-Please discuss the stepwise pattern in asymmetric FBRAs*
Answer: Done (p.18).

*-Please discuss the relationship with tonotopy.*
Answer: Done (p. 13).

----

*Reviewer #2: In this study, Gilday et al use the Ricker-wavelet model (Mexican-hat) to parsimoniously explore bandwidth tuning, sideband inhibition and edge detection in the auditory cortex of awake mice. The authors use a 2D stimulus space to investigate the interactions of center frequency and bandwidth. Based on extracellular recordings across several auditory fields, the authors show a variety of complex trends in frequency-bandwidth response areas (FBRAs) from a population of neurons. To elaborate on some salient features of the data, they use the Ricker-wavelet framework to model FBRAs by manipulating 4 parameters. One of the limitations of the Ricker-wavelet model implemented in the study is that it assumes neural architectures that do not fit with current knowledge of cortical connectivity. As a result, the field of computational vision has largely moved on from the Ricker-wavelet model in order to incorporate recent discoveries of visual cortical architecture and function (e.g. Angelucci et al, Annu Rev Neurosci 2017). The Gilday et al study could potentially be a resource to auditory computational neuroscientists if it incorporated a more modern biological foundation, including new findings on auditory connectomics and function. Another suggestion to enhance the impact of the study would be to relate the findings to functional maps of the areas they are studying (e.g. auditory subfields, tonotopy, etc). Lastly, I suggest that the authors make a greater effort to discuss how their work extends what is already known about surround suppression in the auditory cortex, in particular Zhang et al 2003 (Science). Using conductance measurements of synaptic input in vivo, that study provided numerous insights on side-band inhibition, asymmetries and how they relate to functional maps.*
Answer: We realize that our model is a simple feedforward summation model that makes no explicit assumptions about complex cortical connectomics. Despite the limited set of stimuli that we used, the single motif of side-band inhibition provides a very powerful account for the neuronal responses. Accounting for precise connectomics or the relative contributions of the side band motif versus the various complex spectro-temporal features is beyond the scope of this paper.
We now added a paragraph in the Discussion relating our findings to those of Zhang et al. 2003 (p.14). In addition, we now mention in the limitations section of the Discussion that due to a narrow range of preferred frequencies in our data we were not able to connect our findings to functional maps.
